# Establishment and characterization of an orthotopic patient-derived Group 3 medulloblastoma model for preclinical drug evaluation

**DOI:** 10.1038/srep46366

**Published:** 2017-04-18

**Authors:** Emma Sandén, Cecilia Dyberg, Cecilia Krona, Gabriel Gallo-Oller, Thale Kristin Olsen, Julio Enríquez Pérez, Malin Wickström, Atosa Estekizadeh, Marcel Kool, Edward Visse, Tomas J. Ekström, Peter Siesjö, John Inge Johnsen, Anna Darabi

**Affiliations:** 1 Lund University, Faculty of Medicine, Department of Clinical Sciences Lund, Neurosurgery, Lund, Sweden; 2 Karolinska Institutet, Department of Women´s and Children´s Health, Childhood Cancer Research Unit, Stockholm, Sweden; 3 Uppsala University, Department of Immunology, Genetics and Pathology, Uppsala, Sweden; 4 Karolinska University Hospital, Solna, Center for Molecular Medicine, and Karolinska Institutet, Department of Clinical Neuroscience, Stockholm, Sweden; 5 German Cancer Research Center DKFZ, Division of Pediatric Neurooncology, Heidelberg, Germany; 6 Lund University, Skane University Hospital, Department of Clinical Sciences Lund, Neurosurgery, Lund, Sweden

## Abstract

Medulloblastomas comprise a heterogeneous group of tumours and can be subdivided into four molecular subgroups (WNT, SHH, Group 3 and Group 4) with distinct prognosis, biological behaviour and implications for targeted therapies. Few experimental models exist of the aggressive and poorly characterized Group 3 tumours. In order to establish a reproducible transplantable Group 3 medulloblastoma model for preclinical therapeutic studies, we acquired a patient-derived tumour sphere culture and inoculated low-passage spheres into the cerebellums of NOD*-scid* mice. Mice developed symptoms of brain tumours with a latency of 17–18 weeks. Neurosphere cultures were re-established and serially transplanted for 3 generations, with a negative correlation between tumour latency and numbers of injected cells. Xenografts replicated the phenotype of the primary tumour, including high degree of clustering in DNA methylation analysis, high proliferation, expression of tumour markers, *MYC* amplification and elevated *MYC* expression, and sensitivity to the *MYC* inhibitor JQ1. Xenografts maintained maintained expression of tumour-derived VEGFA and stromal-derived COX-2. VEGFA, COX-2 and c-Myc are highly expressed in Group 3 compared to other medulloblastoma subgroups, suggesting that these molecules are relevant therapeutic targets in Group 3 medulloblastoma.

Medulloblastomas are the most common malignant brain tumours in children. Current standard treatment, including surgery, irradiation and chemotherapy, fail in more than 20% of patients and the long-term adverse effects in survivors are substantial. Transcriptional and epigenetic profiling has described how medulloblastomas can be divided into four molecular subgroups (WNT, SHH, Group 3 and Group 4) with distinct demographics, clinical outcome and implications for targeted therapies^1^. Recently, even further heterogeneity within subgroups has been demonstrated^2^,^3^, and it is clear that an equally heterogeneous supply of relevant experimental models will be needed for the successful development of novel therapeutic approaches.

Established tumour cell lines are extensively used for screening of therapeutic compounds. While patient-derived serum-free brain tumour cultures offers significant advantages over traditionally serum-cultured cell lines^4^, *in vitro* conditions still fail to recapitulate aspects of the tumour microenvironment, such as drug clearance by the circulation, oxygen levels and the impact of non-neoplastic cells on tumour progression. Moreover, the *in vitro* selection pressure generates homogenous cell populations adapted to growth in culture, and extensive cell culturing introduces additional molecular aberrations in the tumour cells^5^. Consequently, the results obtained by drug screenings *in vitro* are only partially predictive for clinical response^6^.

Predisposed genetic mouse models are invaluable tools to study the function of defined mutations in the context of a clinically relevant microenvironment, however these models do not accurately mimic the genetic heterogeneity of primary human tumours. In addition, they usually require complex breeding schemes and may suffer from incomplete tumour penetrance and a variable age of tumour onset. Patient-derived xenograft (PDX) models, generated by inoculation of tumour tissue or low-passage patient-derived tumour cells into immuno-compromised mice, better recapitulate the heterogeneity of the primary tumours, and their molecular fidelity to their human counterparts has been demonstrated for a number of different cancer forms, including brain tumours^7^,^8^,^9^,^10^,^11^. For technical reasons, PDX models are commonly established at subcutaneous sites, but orthotopically implanted tumour cells have been shown to better mimic the drug response, growth pattern and metastatic features of corresponding patient tumours^12^,^13^, likely because of critical influence by the local stroma. Orthotopic inoculation of tumour-derived spheres or fresh surgical samples of glioblastomas^7^,^8^, ependymomas^9^ and medulloblastomas^10^,^11^ has indeed generated brain tumours recapitulating the histology, genotype and phenotype of primary brain tumours.

Among medulloblastomas, Group 3 accounts for ~25% of cases and is associated with the worst prognosis. These tumours are common in younger children, frequently metastatic and less than 50% of patients survive despite aggressive treatment^14^. In contrast to the well-characterized WNT and SHH tumours, the main oncogenic drivers of Group 3 and Group 4 remains to be identified. Although several human cell lines for Group 3 medulloblastoma have been characterized, only few experimental *in vivo* models have been presented of Group 3 and Group 4^15^,^16^. Group 3 tumours harbour few recurrent mutations, but display structural genomic rearrangements (*e.g.* gain of chromosome 1q, loss of chromosome 10q, copy number alterations, tetraploidy and oncogene activation by enhancer hijacking) and epigenetic deregulation^14^,^17^,^18^,^19^,^20^. The most characteristic genetic event is *MYC* amplification, found in ~15% of Group 3 tumours^19^ and identified as a high-risk feature in Group 3 patients^3^.

We have previously described a standardized protocol for establishment and propagation of patient-derived brain tumour cell cultures, either as spheres or monolayers^21^. With this method, we have to date generated cell cultures from >15 primary medulloblastomas, including the clinically aggressive Group 3, MB-LU-181. The surgically removed material displayed >90% proliferation and could be rapidly expanded as spheres. In contrast to non-group 3 medulloblastomas in our cohort, MB-LU-181 cannot be cultured as a monolayer, possibly reflecting the highly aggressive feature of Group 3 tumours^21^. Here, we describe the development and features of a novel orthotopic *MYC*-driven PDX model of Group 3 medulloblastoma, derived from low-passage tumour spheres of MB-LU-181. In addition, we demonstrate increased expression of the relevant therapeutic targets vascular endothelial growth factor A (VEGFA) and cyclooxygenase-2 (COX-2) in Group 3 medulloblastoma patients.

## Results

### Establishment of a transplantable cerebellar Group 3 medulloblastoma model

**Figure 1:**
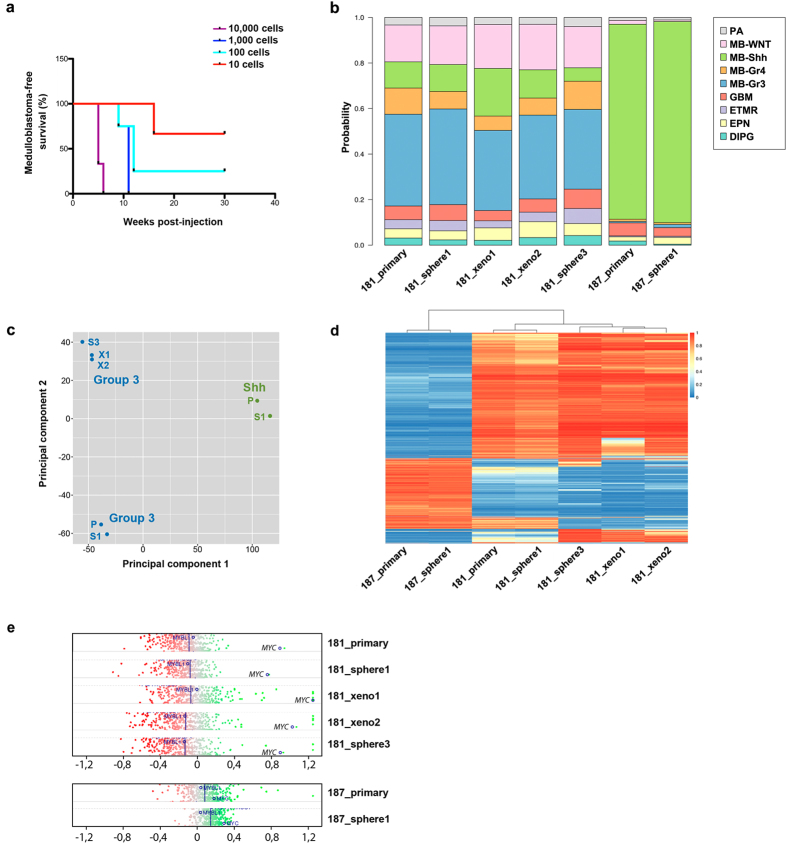
Establishment of medulloblastoma xenografts with maintained methylome. (**a**) Survival curves of NOD-*scid* mice orthotopically inoculated with 10, 100, 1000 and 10,000 medulloblastoma cells (*n* = 3 respectively) isolated from second-generation MB-LU-181 xenografts. An inverse correlation was seen between tumour latency and numbers of cells. (**b**) Genome-wide methylation analysis of primary MB-LU-181, corresponding neurospheres and xenografts displayed a methylation pattern matching to Group 3 medulloblastoma. Primary tissue and corresponding neurospheres from MB-LU-187 displayed a methylation pattern matching to Shh medulloblastoma. MethPed classifications show the probability of a tumour sample belonging to a given group of paediatric brain tumours. Probabilities are shown along the y-axis. PA, pilocytic astrocytoma; MB-WNT, WNT medulloblastoma; MB-Shh, Shh medulloblastoma; MB-Gr3, Group 3 medulloblastoma; MB-Gr4, Group 4 medulloblastoma; GBM, glioblastoma multiforme; ETMR, embryonal tumours with multilayered rosettes; EPN, ependymoma; DIPG, diffuse intrinsic pontine glioma. (**c**) Principal component analysis plot based on normalized beta values, demonstrating the variability across samples in terms of methylation pattern. (**d**) Heatmap representation of unsupervised clustering of DNA methylation profiles in MB-LU-181 (primary tumour, neurospheres and xenografts) and MB-LU-187 (primary tumour and neurospheres). Rows represent the 10.000 probes with highest standard deviations; columns represent samples. DNA methylation levels (normalized beta values) are represented along a color scale ranging from blue to red where blue indicates low methylation level and red indicates high methylation level. (**e**) Copy number analysis showing *MYC* amplification in MB-LU-181 (primary tumour, neurospheres and corresponding xenografts) but not in MB-LU-187 (primary tumor and neurospheres).

Initially, medulloblastoma tissue from MB-LU-181 was sub-grouped as Group 3 using the Illumina HumanMethylation450 array^22^. Low-passage tumour spheres derived from primary MB-LU-181 were orthotopically injected into NOD*-scid* mice and generated tumours with a latency of 17–18 weeks. Sphere cultures were re-established and serially transplanted for 3 generations, with a negative correlation between tumour latency and numbers of injected cells (Fig. 1a). Notably, inoculation of 1000 cells was enough to ensure 100% tumour penetrance within 10 weeks in third generation xenografts and one mouse developed medulloblastoma when ortothopically inoculated with only 10 MB-LU-181 cells (Fig. 1a).

### Epigenetic, histologic and phenotypic profiling of transplanted medulloblastoma cells

Genome-wide methylation assays of primary MB-LU-181 medulloblastoma and the corresponding neurospheres and xenografts resembled Group 3 medulloblastoma (Fig. 1b). As a control, primary medulloblastoma (MB-LU-187) and corresponding neurospheres clustered together and were classified as a Shh medulloblastoma (Fig. 1b). Using principal component analysis, the methylation of the primary samples, corresponding neurospheres and xenograft samples clustered together within subgroups, indicating a strong degree of stability between primary tumours, neurospheres and xenografts (Fig. 1c). Similarly, unsupervised hierarchal clustering also suggests a high degree of relationship between the primary tumour and corresponding neurosphere and xenografts (Fig. 1d). The most evident differences in genome-wide methylation were found between the Group 3 and Shh samples (Fig. 1c,d). The methylation pattern between the primary tumor and first neurosphere culture only exhibited minor changes, whereas, although similar, a more pronounced variation in the methylation pattern was observed between the primary tumor, the serial xenografts and third generation neurospheres (Fig. 1c,d). Copy number variant analysis of genome-wide methylation data showed *MYC* amplification in the primary tumor that was conserved in neurospheres cultures and xenograft transplantations (Fig. 1e; Supplementary Fig. 1).

**Figure 2:**
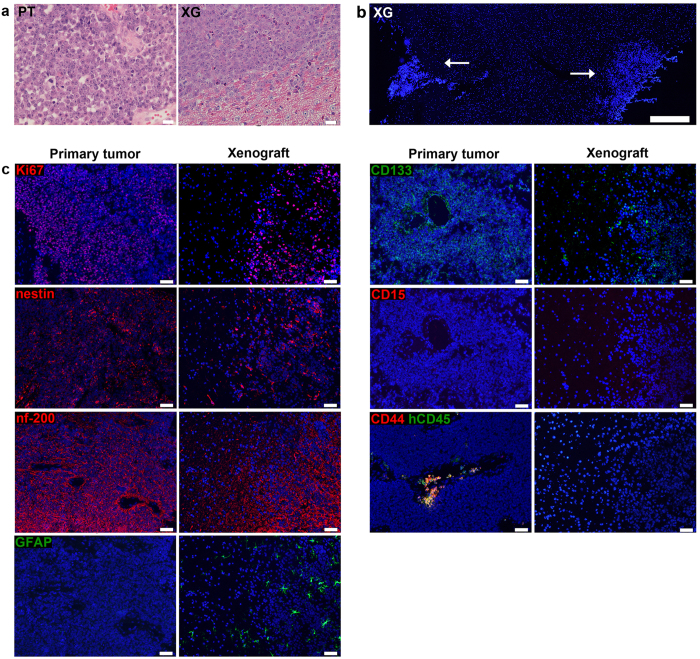
Xenografted medulloblastoma cells maintain the phenotype of the primary tumour. (**a**) H&E-staining of FFPE tissue shows that MB-LU-181 xenografts replicate medulloblastoma histology, including high cellularity, dark staining of round or oval nuclei, sparse cytoplasm and lack of cytoplasmic differentiation. PT, primary tumor; XG, xenograft. Scale bars are 20 μm. (**b**) Overview image of a DAPI-labelled cryosection shows tumor formation in two distinct regions of the cerebellum (*arrows*). Scale bar is 500 μm. (**c**) By immunofluorescent labelling of cryosections of primary medulloblastoma and its derived xenografts, protein positivity is demonstrated for Ki67, nestin and nf-200 in both primary tumour and xenograft. Mouse GFAP astrocytes are detected in the xenografts, while the medulloblastoma cells in primary tumour and xenografts are negative for GFAP. Both primary tumour and xenograft are positive for CD133 and negative for CD15. CD44^+^ cells are seen in areas with CD45^+^ cells in the primary tumour. Xenografts are negative for CD44 and do not contain human CD45^+^ cells. Scale bars are 50 μm.

Xenografts were found to replicate medulloblastoma histology, with a homogenous dense mass of tumour cells displaying a high nuclei-to-cytoplasmic ratio (Fig. 2a) and high proliferation as determined by Ki-67 labelling (Fig. 2c). In addition to the main tumour bulk, xenografted cells were sometimes seen lining the cerebellum and forming smaller secondary tumours (Fig. 2b). To determine if xenografts maintained the protein phenotype of the original tumour, we used immunofluorescent labelling of cryosections to evaluate the expression of a panel of lineage (nestin, nf-200 and GFAP) and putative tumour progenitor/stem cell markers (CD15, CD44 and CD133) in xenografts alongside primary tumour tissue (Fig. 2c). Xenografted cells replicated the phenotype of the original tumour, displaying high expression of nestin, nf-200 and CD133, and no expression of CD15, CD44, GFAP. We have also previously shown that expression of the neuronal progenitor marker CD24^22^ is maintained in xenografts. Although medulloblastoma cells in both primary tumour and xenografts were GFAP-negative, xenografts were infiltrated by star shaped astrocytes that were interpreted as resident mouse astrocytes stained due to species cross-reactivity by the GFAP-antibody. The primary tumour was CD44-negative on tumour cells, but stained positive in clusters of CD45^+^ immune cells.

**Figure 3:**
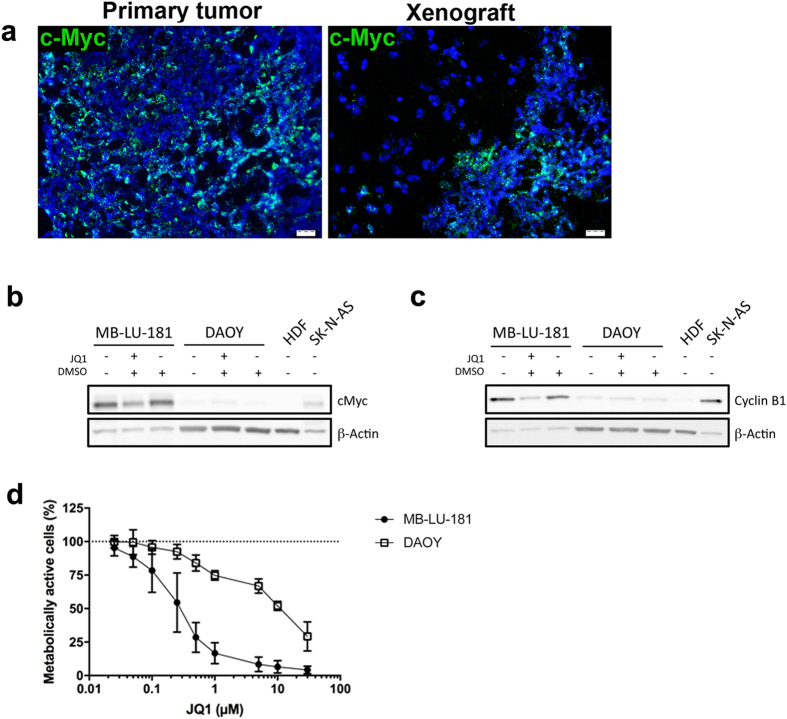
Effect of MYC inhibition in patient-derived Group 3 medulloblastoma. (**a**) *MYC* expression is demonstrated in primary tumour and xenografts with *in situ* hybridization. Scale bar is 20 μm. Western blotting detecting c-Myc (**b**) and cyclin B1 (**c**) in protein extracts isolated from MB-LU-181 cells JQ1 treatment. SK-N-AS neuroblastoma cells and human dermal fibroblasts (HDF) cells were included as positive and negative control for c-Myc expression, respectively. β-actin was used to ensure equal sample loading. (**d**) Cell viability measurement using WST-1 of (**d**) MB-LU-181 and DAOY cells treated with the indicated concentrations of JQ1 for 72-hours. IC_50_ values between MB-LU-181 and DAOY cells were significantly different (*p* < 0.0001 t-test; 0.27 μM in MB-LU-181 versus 10 μM in DAOY). Mean with SD from three independent experiments are displayed.

Both primary tumour and xenografts displayed high levels of *MYC* RNA (Fig. 3a) and MB-LU-181 neurospheres expressed high levels of c-Myc protein compared to DAOY medulloblastoma cells and human dermal fibroblasts cells (Fig. 3b,c). MB-LU-181 neurospheres were also 37-fold more sensitive to treatment with the bromodomain and extraterminal (BET) small molecule inhibitor, JQ1, which indirectly suppresses *MYC* transcription^23^, compared to DAOY cells not expressing *MYC* (IC_50_ 72 h = 0.27 μM for MB-LU-181 and IC_50_ 72 h = 10 μM for DAOY cells) (Fig. 3d). JQ1 treatment was accompanied by c-Myc and cyclin B protein down-regulation in MB-LU-181 cells (Fig. 3b,c).

### Characterization of the microenvironment in medulloblastoma xenografts

**Figure 4:**
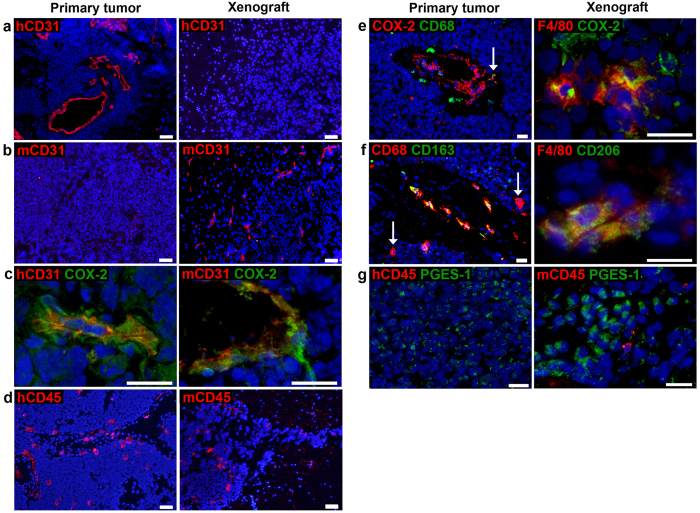
Characterization of the microenvironment in medulloblastoma xenografts. MB xenografts contain (**a**) no human blood vessels (hCD31, red), but (**b**) murine vessels (mCD31, red). In contrast, blood vessels in the primary tumour express (**a**) hCD31, but not (**b**) mCD31. (**c**) Vessel-associated COX-2 (green) is found in both primary tumour and xenograft. (**d**) Primary tumor and xenograft are infiltrated with human (hCD45, red) and murine (mCD45, red) immune cells respectively. Subsets of macrophages (CD68, green/red) in the primary tumour co-expressed (**e**) COX-2 (red), and (**f**) CD163 (green). Both single- and double-positive cells are depicted; *arrows* indicate double-positive cell in (**e**) and single-positive cells in (**f**). Murine myeloid cells (F4/80, red) in MB xenografts express (**e**) COX-2 (green) and (**f**) CD206 (green). (**g**) Tumor cells (not expressing CD45, red) express mPGES-1 (green) in primary tumor and xenograft. Scale bars are 50 μm in **a-b**, **d**, **e** (*to the left)* and f (*to the left)*, and 20 μm in remaining images.

The contribution of the microenvironment to tumour progression is well established, and the lack of human stroma consequently limits the experimental utility of culture-derived xenograft models. To some extent, host endothelial and immune cells may however replace functions of the human stroma. We characterized the vascular and inflammatory cell compartments of medulloblastoma xenografts by immunofluorescent staining of cryosections. Xenografts were devoid of human immune cells (hCD45^+^, Fig. 2c) and human endothelial cells (hCD31^+^, Fig. 4a), consistent with our observation that stromal cells do not survive following serial passaging in sphere-generating cell culturing conditions^21^. Instead, xenografts contained vast numbers of murine blood vessels (mCD31^+^, Fig. 4b). CD31^+^ staining was entwined or lined with inducible nitric oxide synthase (iNOS) and cyclooxygenase-2 (COX-2) in both primary tumour and xenografts (representative images of CD31/COX-2 are shown in Fig. 4c).

NOD-*scid* mice lack mature B and T cells, whereas innate immunity is functional albeit low in activity. Occasional NK cells (NK1.1^+^) and granulocytes (Ly6G/Ly6C^+^) could be detected in the normal compartments of mouse brains, but these cell types were not seen in close proximity to tumours (not shown). In contrast, xenografts were infiltrated with mouse myeloid cells (mCD45^+^, F4/80^+^, Fig. 4d–f) displaying markers indicative of a suppressive phenotype (iNOS^−^, not shown; COX-2^+^, CD206^+^, Fig. 4e,f). The same phenotype was observed in vessel-associated myeloid cells detected in the cerebellum of naïve NOD-*scid* (not shown). In comparison, only a subset of macrophages (CD68^+^) in the primary tumour displayed putative M2 markers (CD163^+^ or COX-2^+^) (Fig. 4e,f). CD8^+^ T cells were detected in or in close proximity to vessels in the primary tumour, but were rarely infiltrating cell dense tumour tissue (not shown).

We have previously described expression of COX-2 in primary medulloblastoma tissues and patient-derived cell cultures, and demonstrated that COX-2 inhibition reduces the growth of subcutaneous medulloblastoma xenografts^21^,^24^. In MB-LU-181 xenografts, COX-2 was predominantly stromal-associated (as exemplified in Fig. 4c,e), whereas the primary tumour MB-LU-181 also displayed areas of tumour cell-associated COX-2 staining in addition to staining on vessels and subsets of macrophages. To investigate the prerequisites for prostaglandin production *in vivo*, we also included microsomal prostaglandin E synthase-1 (mPGES-1), a downstream enzyme of COX-2, in the immune marker panel. mPGES-1 was detected in large clusters of the medulloblastoma cells of primary tumour and xenografts (Fig. 4g), but not in the COX-2^+^ stromal cell compartments.

**Figure 5:**
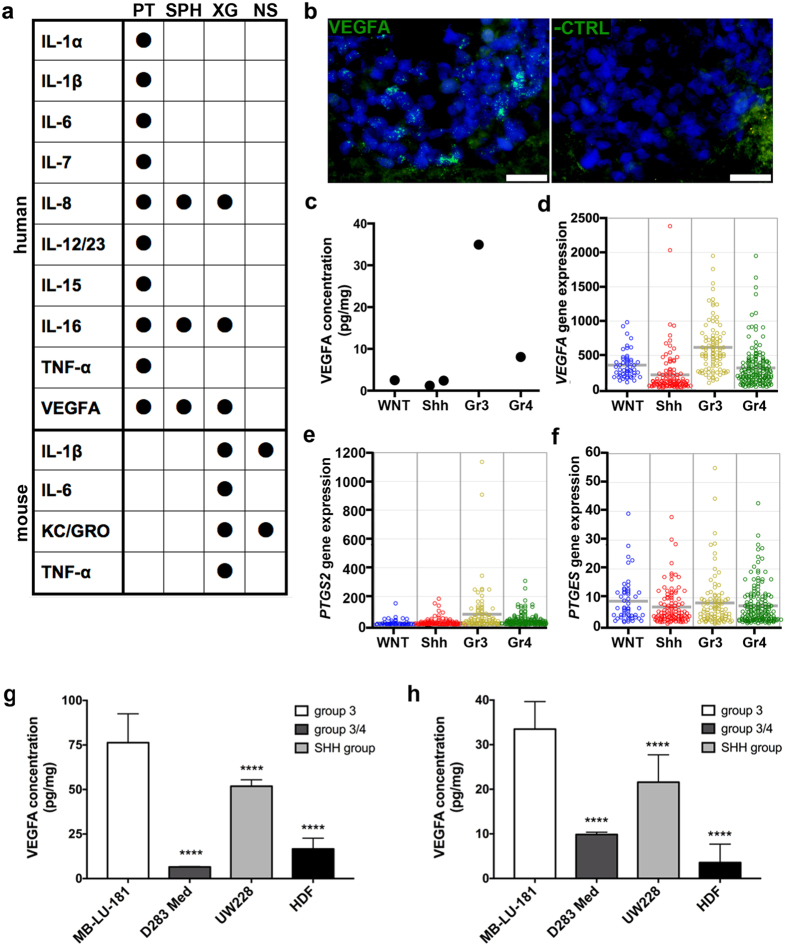
Inflammatory factors in primary tumour, patient-derived sphere cultures and xenografts. (**a**) A panel of inflammatory factors was analysed with MesoScale arrays. Values above LLOQ are indicated with a black dot. PT, primary tumour; XG, xenograft; SPH, cultured spheres; NS, NOD-*scid* cerebellum. (**b**) VEGFA is expressed in xenografted medulloblastoma cells, demonstrated by *in situ* hybridization. To the left, VEGFA probe; to the right, negative control probe on parallel section. Scale bar is 20 μm. (**c**) Mesoscale analysis of VEGFA protein levels in 5 primary medulloblastoma tissues. Median of duplicate values is shown. Gene expression levels of (**d**) *VEGFA*, (**e**) *PTGS2* (COX-2) and (f) *PTGES* (mPGES-1) in medulloblastoma patients (WNT, *n* = 53; Shh, *n* = 112; Group 3, *n* = 94; Group 4; *n* = 164). VEGFA expression in (**g**) supernatants and (**h**) cell extracts from MB-LU-181, D283 Med (group 3/4), UW228 (SHH group) and human dermal fibroblast (HDF) cells were analysed with VEGFA-ELISA. Significant differences compared to MB-LU-181 are marked by asterisks (*p* < 0.0001 (****); one-way ANOVA with Bonferroni as post-test).

The cytokine content of xenografts, alongside primary tumour and cultured spheres, was analysed on antibody-based multiarray platforms detecting 20 human and 10 mouse cytokines respectively. The primary tumour exhibited a wide range of both immune stimulatory and suppressive cytokines (Fig. 5a). Of the 10 analysed mouse-derived factors, 4 were detected in xenografts (Fig. 5a). IL-1β and KC/GRO were found in both xenografts and non-tumour bearing NOD-*scid*, whereas IL-6 and TNF-α were exclusively detected in xenograft tissue.

In cultured medulloblastoma spheres, only IL-8, IL-16 and VEGFA were detected. The secretion of these cytokines was maintained in the xenograft tissue. Expression of VEGFA in xenografted medulloblastoma cells was further confirmed with *in situ* hybridization (Fig. 5b).

Interestingly, we found that the tissue level of VEGFA in MB-LU-181 (depicted as Group 3 in graph) greatly exceeded VEGFA levels in 4 primary medulloblastomas of other molecular subgroups that were analysed in parallel (Fig. 5c). To investigate if the VEGFA observation has clinical relevance, we screened a dataset of 423 medulloblastomas for expression of *VEGFA*, and similarly found that *VEGFA* was preferentially expressed in Group 3 medulloblastomas compared to other subgroups (Anova *p* = 1.7e-17, Fig. 5d). We screened the same cohort for *PTGS2* (COX-2) expression, and similarly found the highest expression in Group 3 medulloblastomas (Anova *p* = 1.2e-06). The expression of *PTGES* (mPGES-1) was similar in all subgroups (Anova *p* = 0.24) (Fig. 5e,f). Also, high levels of VEGFA were detected in both cells and supernatants from MB-LU-181 neurospheres compared to human dermal fibroblast (HDF) cells and cell lines from other medulloblastoma subgroups (Anova, p < 0.0001 for both cells and supernatants) (Fig. 5g,h).

## Discussion

In this study, we describe the development and features of an orthotopic PDX model of Group 3 medulloblastoma (MB-LU-181), intended for future drug evaluation. The aggressive primary tumour displayed high-risk features, including overexpression of *MYC* and >90% cell proliferation, and exhibited rapid clinical progression with <220 days between clinical presentation and death of disease. Its derived xenografts largely recapitulated the epigenetic and phenotypic characteristics of medulloblastoma and maintained high proliferation rate, enabling serial tumour formation from a surprisingly low number of inoculated cells. In addition, xenografts maintained expression of VEGFA and stromal-derived COX-2. Both factors were overexpressed in Group 3 compared to other medulloblastoma subgroups, suggesting these factors to be relevant therapeutic targets for patients with Group 3 medulloblastoma.

To establish the PDX model, we inoculated low-passage tumour spheres into the cerebellum of NOD*-scid* mice. It has been suggested that NSG (NOD*-scid Il2rg*^*null*^) mice should allow for higher tumour penetrance than NOD*-scid* mice, due to lack of NK cell activity^25^. However, we only occasionally detected NK cells in the brains of xenografted NOD-*scid* mice, suggesting that these cells are not actively involved in tumour rejection in this setting. Additional strategies previously used for generating orthotopic brain tumour PDX models include brain inoculation of fresh tumour material, either as a single-cell suspension or tissue chunks. All three methods have resulted in successful establishment of relevant brain tumour models^9^,^12^,^26^, although there is a lack of comparative studies indicating which method should be preferred. For logistic and financial reasons, freezing of tumour material prior to mouse inoculation is commonly required while awaiting histologic or molecular tumour classification. Here, we stored single-cell suspensions at −80 °C for approximately six months before primary xenograft establishment, and briefly cultured thawed tumour cells to ensure viability and active proliferation.

MB-LU-181 xenografts replicated the phenotype of the primary tumour, including histology, expression and amplification of *MYC* and presence or absence of distinct tumour markers. The Group 3 medulloblastoma methylation pattern remained stable throughout serial culturing and xenografting. The highest degree of variation in the genome-wide methylation pattern was observed between the primary tumour and xenografts and corresponding neurosphere derived from the xenograft tumours. This was probably due to the transfer of human tumour cells to a mouse cellular niche, which necessitates adaption of the human cells to a mouse microenvironment. However, these findings are similar to the demonstration that medulloblastomas, including the Group 3 subtype, preserve their methylation subgroup pattern between primary and metastatic compartments^27^. The neurospheres isolated from xenografted tumours exhibited similar epigenetic and phenotypic characteristics compared to the primary tumour, and the neurospheres were highly sensitive to treatment with the *MYC* inhibitor JQ1.

Interestingly, in the xenografted tumours we observed medulloblastoma cells lining the cerebellum and forming secondary tumours distant from the primary tumour, corroborating reports of maintained migratory capacity of medulloblastoma cells following orthotopic injection^28^. Since our primary tumour sample was obtained at a time when the patient not yet displayed evidence of established metastases, our model may mimic the early events in metastatic dissemination that eventually led to fatal relapse in the patient. In the study by Dietl *et al*., the spread of xenografted Group 3 medulloblastoma cells was not mimicked by other transplanted models, suggesting that the migratory features of orthotopic Group 3 models reflects biological behaviour, rather than *e.g.* CSF spread during cell inoculation. Additional studies will be conducted to search for extracranial metastases in the MB-LU-181 model.

In a comprehensive comparison of soluble factors in tumour and xenograft tissue, a range of human pro- and anti-inflammatory factors was detected in the primary medulloblastoma, whereas only IL-8, IL-16 and VEGFA were seen in cultured cells and xenografts, suggesting that most cytokines in the primary tissue were stromal-derived. IL-8 has been attributed both immunosuppressive, pro-angiogenic and direct growth-promoting functions in brain tumours (as reviewed by Brat *et al*.^26^). The lack of conserved IL-8 signalling between mice and human^29^ indicates that the maintained IL-8 secretion in xenografts is not related to tumour/stroma interactions. Rather, IL-8 may play a role as a tumour-specific autocrine or paracrine factor that promotes medulloblastoma proliferation or survival, as previously demonstrated for other brain tumour types^30^,^31^. The role of IL-16 in brain tumours has not been elucidated, although occasional reports have described IL-16 secretion by myeloid cells in astrocytic tumours^32^. Our findings suggest that IL-16 also can be produced by tumour cells. Expression of IL-16 has been described in several peripheral cancers such as breast and prostate cancer^33^,^34^; mechanistic studies are scarce, but it can be speculated that IL-16 contribute to inflammation at tumour sites, for instance by recruitment of leucocytes^33^,^35^.

A critical limit of PDX models is the lack of human stroma, and detailed knowledge about the microenvironment of each individual model is therefore crucial for evaluation of drug strategies affecting tumour-stromal interactions. In the current study, we initially expanded primary tumour cells in sphere-generating culturing conditions, in which non-neoplastic human cells are rapidly out-sorted^21^. Alternative approaches to generate PDX models include injection of fresh surgical specimen, which initially preserves the human stromal compartments. However, this strategy is not feasible for serial passaging, since human stromal cells of the primary xenograft are replaced by its murine counterparts after one or a few passages *in vivo*^11^,^36^,^37^.

Experimental data from us and others^11^ show that the murine vasculature is sufficient to sustain growth of xenografted medulloblastoma cells in the absence of human endothelial cells. The high expression of VEGFA in xenografts and neurospheres indicates that it is one of several potential conserved signalling factors that could direct angiogenesis in this model. VEGF is a key mediator of the hypoxic response in tumours, and VEGFA is one of several angiogenic factors responsible for neovascularization and growth of medulloblastoma^38^. Our results from a gene expression analysis of 423 medulloblastomas further demonstrates enhanced expression of VEGFA in Group 3 compared to other medulloblastoma subgroups, indicating that this patient group in particular may benefit from VEGF-targeted therapies. So far, anti-VEGF compounds have shown limited clinical success in the treatment of adult and paediatric brain tumours, tentatively due to *e.g.* compensatory or parallel angiogenic mechanisms, skewed patient selection or the use of antibodies directed against both anti- and pro-angiogenic VEGF isoforms^39^. Nevertheless, targeting of angiogenic pathways remains a promising treatment approach for brain tumour patients in combination with other treatment strategies such as cytotoxic agents^40^. In this context, PDX models enable detailed *in vivo* studies of angiogenic signalling and treatment resistance^41^, as well as the subsequent design of more specific drug strategies that are optimal for distinct tumour subsets.

In addition to VEGF, the COX-2/mPGES-1/PGE_2_ pathway has been investigated as a therapeutic target for a number of cancer forms, including paediatric brain tumours^24^. COX-2 is the rate-limiting enzyme for production of PGE_2_ via the terminal enzyme mPGES-1. PGE_2_ has been attributed a wide range of tumour-promoting functions, including increased survival, proliferation, invasiveness and chemo-resistance of tumour cells, as well as potent inhibition of anti-tumour immune effector functions^42^,^43^. We have previously shown that inhibition of COX-2 activity can reduce the growth of subcutaneous neuroblastoma and medulloblastoma PDXs^24^,^44^, and the enhanced expression of COX-2 in patients with Group 3 medulloblastoma suggest that therapeutic intervention may be particularly useful for this patient group. Several studies have demonstrated a direct link between COX-2 and VEGF signalling in tumour cells. PGE_2_ may induce the expression of VEGF and thereby contribute to the angiogenic response and subsequent tumour evasion^45^,^46^. Conversely, VEGF may induce COX-2 expression in endothelial cells^47^. More recently, it was suggested that VEGF and COX-2 could act as independent regulators of angiogenesis, and that expression of COX-2 facilitates VEGF activity^48^. Consequently, COX-2 expression may partly be responsible for tumour resistance to VEGF-therapies, and combined inhibition of VEGF and COX-2 is a possible treatment strategy that may of particular interest for Group 3 medulloblastoma patients.

Interestingly, we found that distinct cell populations were responsible for the production of COX-2 and mPGES-1 in xenografts, where COX-2 was vessel- and macrophage-derived, while mPGES-1 detection was restricted to tumour cells. The primary tumour displayed the same staining patterns, although COX-2 was also seen on subsets of tumour cells. We have repeatedly observed distinct cellular origin of mPGES-1 and COX-2 in neuroblastoma^44^, experimental glioma^49^ and here in medulloblastoma, and demonstrated that inhibition of COX-2 has a therapeutic effect *in vivo* even though COX-2 and mPGES-1 are expressed in different cell types^49^. These data suggests that intermediate metabolites are transferred between cell types, as previously described^50^, and the MB-LU-181 model will be useful to study such interactions. Still, it has to be confirmed that PGE_2_ is indeed produced in xenografts.

Human medulloblastomas generally exhibit a quiescent or suppressed immune cell response compared to other paediatric brain tumour types, including few T cells and myeloid cells and a general lack of immune activation^51^. Immunodeficient mouse models may therefore better mimic the microenvironment of medulloblastomas than of brain tumour types with a more prominent immune activation. The most frequent immune cells detected in both primary tumour and xenografts were myeloid cells. Based on established markers of myeloid polarization, xenograft-infiltrating mouse myeloid cells displayed a suppressed/inactivated phenotype, reminiscent of myeloid cells detected in naïve NOD-*scid*. Even so, tumour-bearing mice displayed an up-regulation of IL-6 and TNF-α, which are key pro-inflammatory cytokines in macrophage activation^52^ – suggesting that murine macrophages indeed have the functional capacity to respond to inflammatory stimuli (either the presence of tumour cells or tissue injury induced by inoculation). It is however unclear if the response is sufficient to have an antitumor effect.

It is also not clear at this point to what extent the murine macrophage response mimics the clinical situation. Cytokine profiling of the primary tumour identified human IL-6 and TNF-α – consistent with the corresponding murine factors found in the mouse tissue, and indicative of pro-inflammatory response – but also a range of other factors that could be derived from both innate and adaptive immune cells. The predominant phenotype of myeloid cells in the primary tumour could not be clearly determined in this study; tentative M2 marker CD163 were absent on most CD68 cells, and a cytokine profiling identified a range of factors that could be derived from pro-inflammatory (IL-1, IL-6, IL-12/23, IL-15, TNF-α) and suppressive (IL-8, VEGFA) cells respectively^53^. Even so, studies of clinical samples and syngeneic mouse models show that Group 3 and Group 4 tumours are associated with low macrophage activity compared to other medulloblastoma subgroups^54^,^55^, and it would therefore be highly relevant to compare our results to matched clinical samples and subgroup-specific PDX models generated in the same experimental setting.

In summary, we describe a novel orthotopic *MYC*-driven PDX model of Group 3 medulloblastoma. The markedly poor outcome for patients with Group 3 medulloblastoma highlights the limits of current treatment protocols and the need for new therapeutic strategies. The PDX model described here represents a rare opportunity to investigate the biology of a particularly aggressive medulloblastoma, and evaluate novel drug strategies for a tumour that proved to be incurable by standard treatment.

## Methods

### Tumour sample used for xenografts

All experiments were performed in accordance with national regulations and were approved by the Local Ethical Review Board of Lund, Sweden (ETIK2008/642) and the Ethical Research Board of the Medical Faculty at Lund University, Lund, Sweden (serial number LU1028-03). All patients and/or their parents gave their informed consent prior to inclusion in the study. Tumour tissue was collected from a 4-year-old male medulloblastoma patient and coded as MB-LU-181. Tumour tissue from resected surgical material was split in four parts that were (a) snap-frozen and stored at −80 °C, (b) fixed in NBF for routine H&E-staining, (c) frozen in liquid N_2_ precooled isopentane (−55 °C, VWR International AB, Lund, Sweden) for cryosectioning, and (d) used to establish a cell culture and subsequently xenografts, see below. Adjacent tumour tissue was sent for routine PAD, where the tumour was diagnosed as a medulloblastoma. MB-LU-181 was initially assigned a Group 3 affiliation by the methylation profile using Illumina Infinium 450k methylation array of snap-frozen tissue, as previously described^22^.

### Establishment of orthotopic medulloblastoma xenografts

All animal experiments were approved by the regional ethics committee for animal research (N137/10), appointed and under the control of the Swedish Board of Agriculture and the Swedish Court. Surgically removed MB-LU-181 tissue was incubated with TrypLE™ Express (Gibco^®^, Life Technologies, Stockholm, Sweden) and mechanically dissociated through a BD Falcon™ cell strainer (75 μm) (BD Biosciences Pharmingen, Stockholm, Sweden). Cells were seeded on Ultra-Low™ 6-well plates (Corning, VWR, Stockholm, Sweden) in UltraCULTURE™ cell culture medium (Lonza BioWhittaker Inc., VWR) supplemented with 2 mM L-glutamine (Lonza BioWhittaker Inc., VWR), 1% antibiotics (Penicillin-Streptomycin, Life Technologies) and EGF (20 ng/ml, Chemicon, Merck Millipore, Solna, Sweden). Following sphere formation, spheres were mechanically passaged and further propagated as previously described^21^. Cells were kept in culture for approximately one week before the first transplantation and for 2–3 weeks between *in vivo* passages, and were passaged approximately once a week.

Immediately prior to *in vivo* transplantation, spheres were dissociated, counted and kept on ice in Hank’s Balanced Salt Solution (VWR). Initially, eight weeks old female NOD*-scid* mice (NOD/MrkBomTac-Prkdcscid) were anesthetized with isoflurane and mounted in a stereotactic frame (David Kopf Instruments). A 6 μl cell suspension containing 20,000 low-passage tumour cells was orthotopically injected into the cerebellum (*n* = 2) at median/lateral + 1.0 mm, anterior/posterior −2.0 mm, dorsal/ventral −2.5 mm relative to lambda, with the needle at 30° using a 26 ga Hamilton syringe (Sigma Aldrich). The needle was left *in situ* for 1 min after lowering the needle and 2 min after the injection of the cell suspension, which was performed with a speed of 2 μl per min. Neurological symptoms and weight loss indicative of brain tumour growth appeared after 17 and 18 weeks, respectively. Mice were then euthanized by carbon dioxide followed by cervical dislocation and cerebellar tissue was dissected into two parts, where one was used to re-establish sphere cultures for serial orthotopic transplantation (using the same protocol as described for the patient cell culture).

### Neurospheres, cell lines and *in vitro* treatments

MB-LU-181 and MB-LU-187^21^ neurospheres were cultured and maintained *in vitro* as previously described in *Establishment of orthotopic medulloblastoma xenograft* section. DAOY cell line (ATCC^®^ HTB186™), SK-N-AS (ATCC^®^ CRL-2137™) and D283 Med (ATCC^®^ HTB-185^TM^) were purchased from the American Type Culture Collection (ATCC, Manassas, USA). UW228 cell line was a kind gift from Prof. Monica Nistér (Karolinska Institutet, Stockholm, Sweden). The Human Dermal Fibroblast (HDF) cell line was kindly provided by Prof. Per-Johan Jakobsson (Karolinska Institutet, Stockholm, Sweden). DAOY cells were cultured in Eagle’s Minimum Essential Medium, SK-N-AS cells in Dulbecco’s Modified Eagle’s Medium, D283 Med in Dulbecco’s Modified Eagle Medium-GlutaMAX 4.5 gr/mL glucose and UW228 in Dulbecco’s Modified Eagle Medium/F12. HDF cells were cultured and maintained in RPMI medium. Media for DAOY, SK-N-AS, UW228, D283 Med and HDF cell lines were supplemented with 2 mM L-glutamine, 1% Penicillin/Streptomycin, 10% Fetal Bovine Serum (FBS) and 1% non-essential amino acids (only for D283 Med). All the media and supplements were purchased from GIBCO (GIBCO, Paisley, UK).

MB-LU-181 spheres isolated from 2^nd^ generation of xenografts, DAOY and HDF cells were treated with JQ1 ((+/−)-JQ1, SIGMA, Saint Louis, USA). JQ1 was dissolved in DMSO and added to the medium at the corresponding volume to obtain the desired final concentrations. All treatments were performed in OPTI-MEM media (GIBCO, Paisley, UK) supplemented with 2 mM L-glutamine and 1% Penicillin/Streptomycin, except for MB-LU-181 neurospheres, where the compound was dissolved in the previous described culturing medium.

### Cell viability assay

Cell viability was measured using WST-1 (ROCHE, Mannheim, Germany) following the manufacturer’s instructions. Briefly, cells were seed in 96-well plates (TPP^®^, Trasadingen, Switzerland) and incubated overnight before the indicated concentrations of JQ1 were added. After 72 h, the WST-1 reagent was added and absorbance was measured. Three independent experiments with 8 replicates per conditions were performed. Data are expressed as relative survival compared to DMSO-treated cells, and IC_50_ values were compared with unpaired t-test. The IC_50_ was determined using non-linear regression analysis on effect-log concentration curves (GraphPad Prism).

### *In vitro* VEGFA measurement

VEGFA was measured *in vitro* using the Human VEGFA ELISA Kit (Thermo Scientific, Rockford, USA) in accordance with the manufacturer’s instructions. Briefly, MB-LU-181 neurospheres were seeded in Corning Cell-Bind 6-well plate (Corning, VWR, Stockholm, Sweden). As a normal reference, HDF cells were seeded in 10 cm plates (Sarstedt, Nümbrecht, Germany) and incubated over night to allow the cells to attach. Then, the medium was replaced by OPTI-MEM and supernatants and pellets were collected after 72 h of incubation. Total protein content from pellets was obtained by lysis with RIPA buffer (Thermo Scientific, Rockford, USA). The protein concentration of pellets and supernatants was determined using the BCA assay kit (Thermo Scientific, Rockford, USA). The VEGFA expression was measured with ELISA and values were normalized to the total protein content. Three independent samples for each cell line and time point were obtained and processed. Values were compared with two-way ANOVA with Bonferroni as post-test.

### Western blotting

Western blot analyses were used to assess protein expression of c-Myc and cyclin B1. Three independent experiments for each cell line and condition were carried out. Total proteins were extracted using RIPA buffer (Thermo Scientific, Rockford, USA) supplemented with Halt^TM^ Protease and Phosphatase Inhibitor Cocktail (Thermo Scientific, Rockford, IL, USA). A total of 20 μg protein was resolved by SDS-PAGE and transferred onto a nitrocellulose membrane. After membrane blocking, the incubation with primary antibody was carried out with c-Myc (D84C12) Rabbit mAb (#5605, Cell Signaling, Danvers, USA) (dilution 1/1000), cyclin B1 Mouse mAb (sc-245, Santa Cruz Biotechnology, Santa Cruz, USA) (dilution 1/500), and β-Actin mouse mAb (#3700, Cell Signaling, Danvers, USA) (dilution 1/10,000), followed by incubation with a peroxidase-conjugated goat anti-rabbit antibody (#7074, Cell Signaling, Danvers, USA) (dilution 1/5,000) or horse anti-mouse (#7076, Cell Signaling, Danvers, USA) (dilution 1/4000 for cyclin B1 and 1/20,000 for β-Actin). Visualization of signal was carried out with an ECL plus chemoluminescence detection system (GE Healthcare, Buckinghamshire, UK). β-Actin level was used as loading control.

### Histology and immunofluorescent labelling of tumour and xenograft sections

NBF-fixed MB-LU-181 tissue and derived xenografts were stained with routine HTX/eosin-staining and histology was evaluated by a trained pathologist. Immunofluorescent labeling of cryosections was performed as previously described^49^. The following primary antibodies were used: mouse anti-CD133/2 (5 μg/ml, Milteney Biotec GmbH, Bergisch Gladbach, Germany); FITC-mouse anti-GFAP (5 μg/ml), PE-mouse anti-human CD8 (diluted 1:10), PE-mouse anti-human CD15 (diluted 1:10), PE-mouse anti-human CD44 (diluted 1:10), FITC-mouse anti-human CD45 (diluted 1:10), FITC-mouse anti-human CD163 (diluted 1:10), rat anti-mouse NK-1.1. (PK136) (10 μg/ml), rat anti-mouse CD45 (5 μg/ml) rat anti-mouse Ly6G/Ly6C (5 μg/ml), rat anti-mouse CD31 (5 μg/ml) (BD Biosciences); rabbit anti-human nestin (5 μg/ml), mouse anti-human CD31 (5 μg/ml), rabbit anti-COX-2 (5 μg/ml), rabbit anti-mannose receptor (CD206) (4 μg/ml) (Abcam, Cambridge Science Park, Cambridge, UK); rabbit anti-Ki67 (diluted 1:40, Biotrend, Köln, Germany); rabbit anti-human neurofilament 200 (5 μg/ml) (Sigma Aldrich); purified rat anti-mouse F4/80 (5 μg/ml), rabbit anti-iNOS (10 μg/ml) (AbD Serotec, Morphosys AbD GmbH, Düsseldorf, Germany); rabbit anti mPGES-1 (1:1000) (Agrisera AB, Vännäs, Sweden); mouse anti-human CD68 (5 μg/ml) (eBioscience, AH diagnostics AB, Skärholmen, Sweden). Alexa Fluor 594-goat anti-rabbit, Alexa Fluor 488-goat anti-rabbit and Alexa Fluor 594-donkey anti-rat (5 μg/ml) (Molecular Probes, Life Technologies) were used as secondary antibody for unconjugated primary antibodies. Matched isotype antibodies were used as negative controls.

### Cytokine profiling of tissue

Cytokine profiling was performed on MB-LU-181 tissue, MB-LU-181-derived spheres and xenograft tissue, normal cerebellar tissue from naïve NOD*-scid* mice, and tissues isolated from 4 other primary medulloblastomas (MB-LU-140, MB-LU-159, MB-LU-207, MB-LU-208; patient details and molecular subgrouping have been previously described in references^21^,^22^ and cytokine values from MB-LU-140 tissue have been previously published in reference^21^). All samples were mechanically homogenized in 1 ml/100 mg MSD Tris Lysis Buffer (Meso Scale Discovery, Rockville, MD, USA), incubated on ice for 20 min and centrifuged for 10 min at 2000 g. Supernatants were collected and stored at −80 °C until subsequent analysis. Protein lysates were analysed in duplicates with high-sensitivity cytokine multiplex assays (Meso Scale Discovery) Mouse Proinflammatory Panel 10-Plex (IFNγ, IL-1β, IL-2, IL-4, IL-5, IL-6, KC/GRO, IL-10, IL-12p70, TNF-α), Human Proinflammatory Panel 1 (IFNγ, IL-1β, IL-2, IL-4, IL-6, IL-8, IL-10, IL-12p70, IL-13, TNF-α) and Human Cytokine Panel 1 (GM-CSF, IL-1α, IL-5, IL-7, IL-12/IL-23p40, IL-15, IL-16, IL-17A, TNF-β, VEGF) according to the manufacturer´s instructions. LLOQ (lower limit of quantification) was used as cut-off for positive detection. To exclude species cross-reactivity of the panels, supernatants of *in vitro*-stimulated human and mouse macrophages (THP-1 and RAW) respectively were analysed in parallel on human and mouse platforms.

### *In situ* hybridisation

*In situ* hybridisation was performed with the RNAscope multiplex fluorescent reagent assay (ACD-320850, Advanced Cell Diagnostics, Hayward, CA, USA) according to the manufacturer’s instructions. Briefly, 10 μm sections of cryosections of primary and xenografted MB-LU-181 were hybridized with target probes (Hs-MYC, 311761; Hs-VEGFA, ACD-423161; positive control, ACD-320861; negative control, ACD-320871; Advanced Cell Diagnostics) and labelled with Alexa 488. Nuclei were counterstained with ProLong Gold anti fading containing DAPI (Molecular Probes, Life Technologies, Stockholm, Sweden).

### Gene expression analysis and genome-wide methylation profiling

Gene expression data from 423 medulloblastomas were obtained from multiple gene expression profiling studies (Kool *et al*.^56^, Fattet *et al*.^57^, Robinson *et al*.^17^, McCabe *et al*. unpublished data and Kool *et al*. unpublished data). All samples were analysed on the Affymetrix GeneChip Human Genome U133 Plus 2.0 arrays. The MAS5.0 algorithm of the GCOS program (Affymetrix Inc) was used for normalization of the expression data and microarray data was visualized and analysed using R2 software (http://R2.amc.nl). Expression of *VEGFA, PTGS2* (COX-2) and *PTGES* (mPGES-1) was compared between subgroups with one-way ANOVA.

Genomic DNA from the primary MB-LU-181 tumour, subsequent generations of sphere cultures, serially transplanted mice was extracted from primary tumour tissue, dissected xenografts, and cultured spheres using GenElute™ Mammalian Genomic DNA Miniprep Kit (Sigma-Aldrich, Sweden) according to the manufacturer’s specifications. The concentration of DNA was measured with Nanodrop. Genome-wide methylation profiling was performed using the InfiniumEPIC BeadChip platform (Illumina, San Diego, California, USA) by the SNP&SEQ Technology Platform in Uppsala, Sweden (www.genotyping.se). DNA from another primary medulloblastoma (MB-LU-187) and its first-generation cultured sphere was also analysed.

Raw IDAT files were imported and analysed using the Bioconductor packages minfi^58^ and RnBeads^59^. Methylation signals were preprocessed and normalized with the *preprocessIllumina* command and data quality was evaluated using the *getQC* feature. Normalized beta values were used to classify the samples using the MethPed algorithm^60^, to generate PCA plots, and to perform unsupervised hierarchical clustering. Copy number profiles are based on the Bioconductor package conumee and were generated using a publicly available methylation profiling tool developed and hosted by the DKFZ and University of Heidelberg (http://www.molecularneuropathology.org).

## Additional Information

**How to cite this article:** Sandén, E. *et al*. Establishment and characterization of an orthotopic patient-derived Group 3 medulloblastoma model for preclinical drug evaluation. *Sci. Rep.* **7**, 46366; doi: 10.1038/srep46366 (2017).

**Publisher's note:** Springer Nature remains neutral with regard to jurisdictional claims in published maps and institutional affiliations.

## Supplementary Material

Supplementary Figure S1
