# Supplementary material for: Establishment and characterization of an orthotopic patient-derived Group 3 medulloblastoma model for preclinical drug evaluation
Source: Sci Rep. 2017 Apr 18;7:46366. doi: 10.1038/srep46366 (PMC5394470; doi:10.1038/srep46366)
Supplement: Supplementary Figure S1 [file srep46366-s1.pdf]

Establishment and characterization of an orthotopic patient-derived Group 3 medulloblastoma model for preclinical drug evaluation

Emma Sandén[1,a], Cecilia Dyberg[2,a], Cecilia Krona[3], Gabriel Gallo-Oller[2], Thale Kristin Olsen[2], Julio Enríquez Pérez[1], Malin Wickström[2], Atosa Estekizadeh[4], Marcel Kool[5], Edward Visse[1], Tomas J. Ekström[4], Peter Siesjö[1,6], John Inge Johnsen[2,b], Anna Darabi[1,b*]

[*]corresponding author

[a]shared first authorship

[b]shared senior authorship

[1]Lund University, Faculty of Medicine, Department of Clinical Sciences Lund, Neurosurgery, Lund, Sweden

[2]Karolinska Institutet, Department of Women´s and Children´s Health, Childhood Cancer Research Unit, Stockholm, Sweden

[3]Uppsala University, Department of Immunology, Genetics and Pathology, Uppsala, Sweden

[4]Karolinska University Hospital, Solna, Center for Molecular Medicine, and Karolinska Institutet, Department of Clinical Neuroscience, Stockholm, Sweden

[5]German Cancer Research Center DKFZ, Division of Pediatric Neurooncology, Heidelberg, Germany

[6]Lund University, Skane University Hospital, Department of Clinical Sciences Lund, Neurosurgery, Lund, Sweden

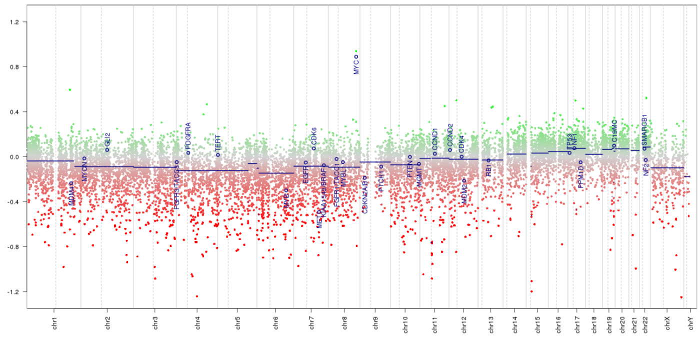

181_primary

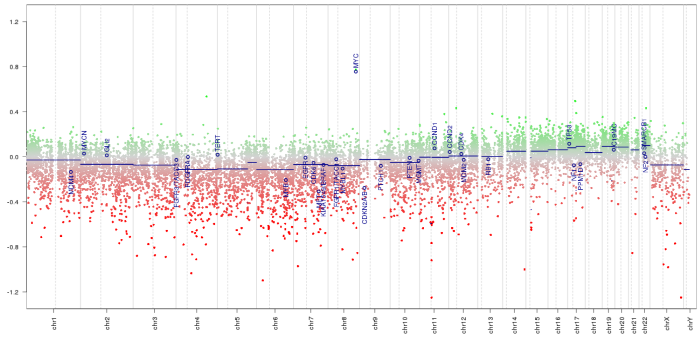

181_sphere1

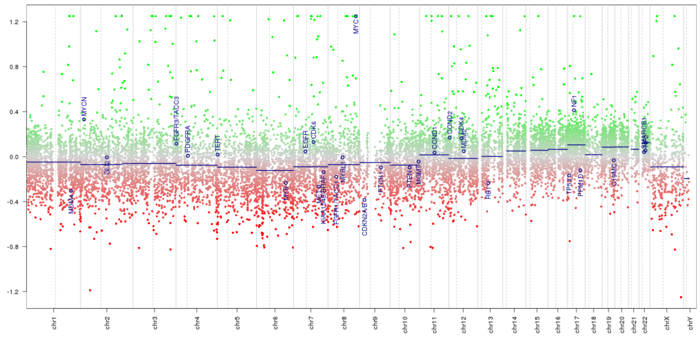

181_xeno1

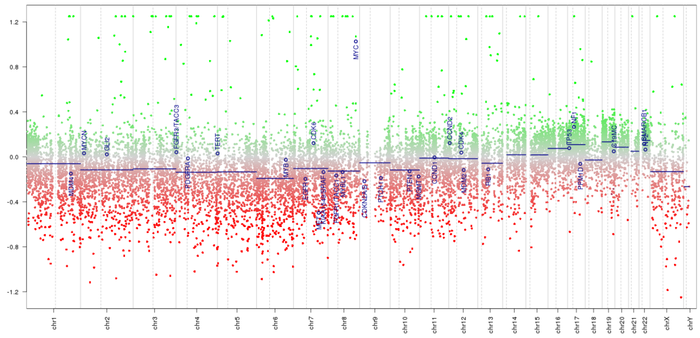

181_xeno2

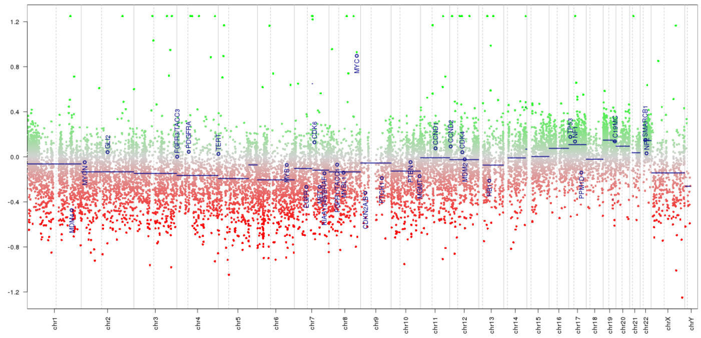

181_sphere3

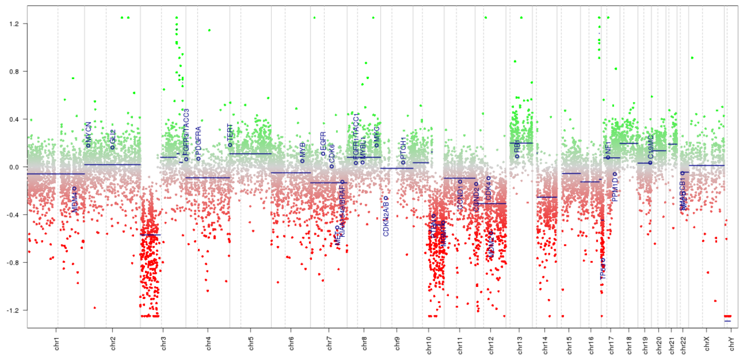

187_primary

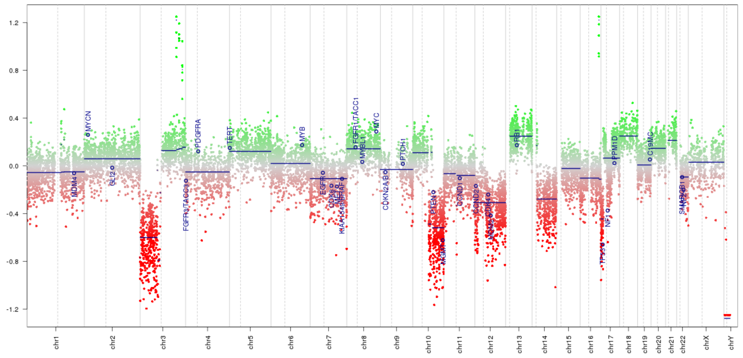

187_sphere1

Supplementary Figure S1. Copy number variance analysis of MB-LU-181 (Group 3 MB; primary tumour, neurospheres and xenografts) and MB-LU-187 (Shh MB; primary tumour and neurospheres).
